# A Janus Adhesive Hydrogel with Integrated Attack and Defense for Bacteria Killing and Antifouling

**DOI:** 10.34133/bmef.0059

**Published:** 2024-10-02

**Authors:** Kai Ren, Xiang Ke, Miao Zhang, Yuan Ding, Hao Wang, Hong Chen, Jing Xie, Jianshu Li

**Affiliations:** ^1^ College of Polymer Science and Engineering, State Key Laboratory of Polymer Materials Engineering, Sichuan University, Chengdu 610065, P.R. China.; ^2^ State Key Laboratory of Oral Diseases, West Chin Hospital of Stomatology, Sichuan University, Chengdu 610041, P.R. China.; ^3^ Med-X Center for Materials, Sichuan University, Chengdu 610041, P.R. China.

## Abstract

**Objective:** Skin wound exposed to complex external environment for a long time is highly susceptible to bacterial infection. **Impact Statement:** This work designs a Janus adhesive dual-layer hydrogel containing in situ silver nanoparticles (named PSAP/DXP@AgNPs) with integrated attack and defense to simultaneously kill the existing bacteria and prevent foreign bacterial contamination. **Introduction:** The current gauze dressing fixed by tape fails to well fit at skin wound and lacks intrinsic antibacterial property, making it highly prone to causing secondary infection. Moreover, foreign bacteria may contaminate the wound dressing during use, further increasing the risk of secondary infection. **Methods:** In this work, a Janus adhesive dual-layer PSAP/DXP@AgNPs hydrogel is prepared by sequentially building the PSAP gel layer containing zwitterionic poly(sulfobetaine methacrylamide) (PSBMA) on the DXP@AgNPs gel layer containing in situ catechol-reduced AgNPs. **Results:** The flexible PSAP/DXP@AgNPs can adapt shape change of skin and adhere to skin tissue with interfacial toughness of 153.38 J m^−2^ relying on its DXP@AgNPs layer, which is beneficial to build favorable fit. The in situ reduced AgNPs released from the DXP@AgNPs layer of PSAP/DXP@AgNPs exhibit obvious antibacterial effects against *Escherichia coli* and *Staphylococcus aureus*, with antibacterial rates of 99% and 88%, respectively. Meanwhile, the hydrated PSAP layer of PSAP/DXP@AgNPs containing PSBMA is able to prevent the bacterial contamination, decreasing the risk of secondary infection. Besides, cell experiments demonstrate that PSAP/DXP@AgNPs is biocompatible. **Conclusion:** The PSAP/DXP@AgNPs hydrogel with integrated attack and defense simultaneously possessing bacteria-killing and bacteria-antifouling properties is a potential alternative in treating infected skin wound.

## Introduction

Skin is the largest exposed organ of the human body and protects other internal organs from damage and microbial invasion [1]. In the Unites States alone, more than 6 million people are affected every year by wounds associated with excess mortality, resulting in direct annual treatment costs in excess of $25 billion [2]. When the skin is injured by external incision, graze, burning, and so on, the wound is inclined to encounter bacterial infection, delaying the healing process [3]. The symptoms of infection including tissue fluid exudation, swelling, warmth, and pain threatening life and health of human even directly cause death in severe cases [4,5]. In the clinic, the common practice for wound sterilization typically involves using either 75% alcohol or iodine solutions, followed by covering with cotton gauze [6]. Due to the lack of intrinsic antibacterial property for cotton gauze, the horrible secondary infection easily occurs during wound healing. In addition, the fit between tape-fixed gauze and skin tissue is not good enough, increasing the risk of secondary infection. Hence, there is an urgent demand within the biomedical field for the development of wound dressing that possesses long-lasting antibacterial property and easy application.

Adhesive hydrogel with an adjustable 3-dimensional structure can not only maintain moist environment and permeability to facilitate metabolism and wound healing but also dispense with auxiliary fixation of biomedical tape [7–10]. Thus, the adhesive hydrogel wound dressing with antibacterial property is a potential alternative for traditional medical gauze [11]. Xu et al. [12] reported that an adhesive hydrogel loaded with tobramycin adhering to wound can kill the invasive bacteria by releasing antibiotic. Meanwhile, the use of antibiotic is likely to increase the bacterial resistance, resulting in declining drug effect. The characteristic of negatively charged bacterial surface offers an antibacterial strategy without worrying about the bacterial resistance. Physically disrupting the structures of bacterial cell wall and membrane by positively charged substance has been widely studied in antibacterial field [13–15]. Guo et al. [16] reported that an adhesive chitosan-based hydrogel dressing exhibited certain antibacterial properties derived from positively charged protonation of amino groups on chitosan. However, the protonation of amino groups dangling onto chitosan is highly susceptible to acidity, leading to an unstable antibacterial property. Silver nanoparticles (AgNPs) as a kind of widely used broad-spectrum antibacterial agent can release positive silver ions to disrupt bacterial cell wall and membrane, denature the microbial proteins, and disturb the DNA replication, finally killing bacteria [17–20]. Ge et al. [21] reported that antibacterial hydrogel dressing with adhesion enabled the release of the doped AgNPs to kill bacteria. The doped AgNPs are liable to ununiformly distribute in hydrogel, resulting in severe aggregation. The penetration of AgNPs and the release of silver ions may be restricted, decreasing antibacterial property [22–24]. In a word, current adhesive hydrogel dressings with an antibacterial property still exhibit some drawbacks that require further improvement.

Bacteria-killing hydrogel wound dressing with double-sided adhesion exposed to complex external environment is prone to be contaminated by foreign bacteria. Once the adhesion between bacteria and hydrogel dressing is built, the bacterial accumulation on the outer surface of hydrogel dressing may further invade the covered wound, leading to secondary infection. In fact, under the assistance of appendages (e.g., flagella and fimbriae), the bacteria relying on its adhesive proteins trigger anchoring to various substrates through noncovalent interactions (including electrostatic interactions, hydrophobic interactions, or van der Waals interactions) [25–27]. Hence, the protein-antifouling property on the outer surface of hydrogel dressing highly determines its bacteria-antifouling property, influencing the bacterial accumulation and risk of secondary infection. The polyethylene glycol (PEG) can provide antifouling property due to the hydration effect induced by hydrogen bond with water molecule [27]. Buxadera-Palomero et al. [28] reported that PEG coating on titanium decreased bacterial contamination by up to 90%. Different from PEG, zwitterionic polymer interacts with water molecules through ion–dipole interaction, providing better hydration effect [27,29]. Smith et al. [30] reported that the zwitterionic poly(sulfobetaine methacrylamide) (PSBMA) coating on catheter could provide robust hydrated shell to reduce bacterial contamination by 96%. Therefore, the construction of the antifouling layer on antibacterial hydrogel dressing with adhesive property is expected to kill the existing bacteria on the wound while simultaneously resisting the secondary infection of foreign bacteria.

**Fig. 1.**
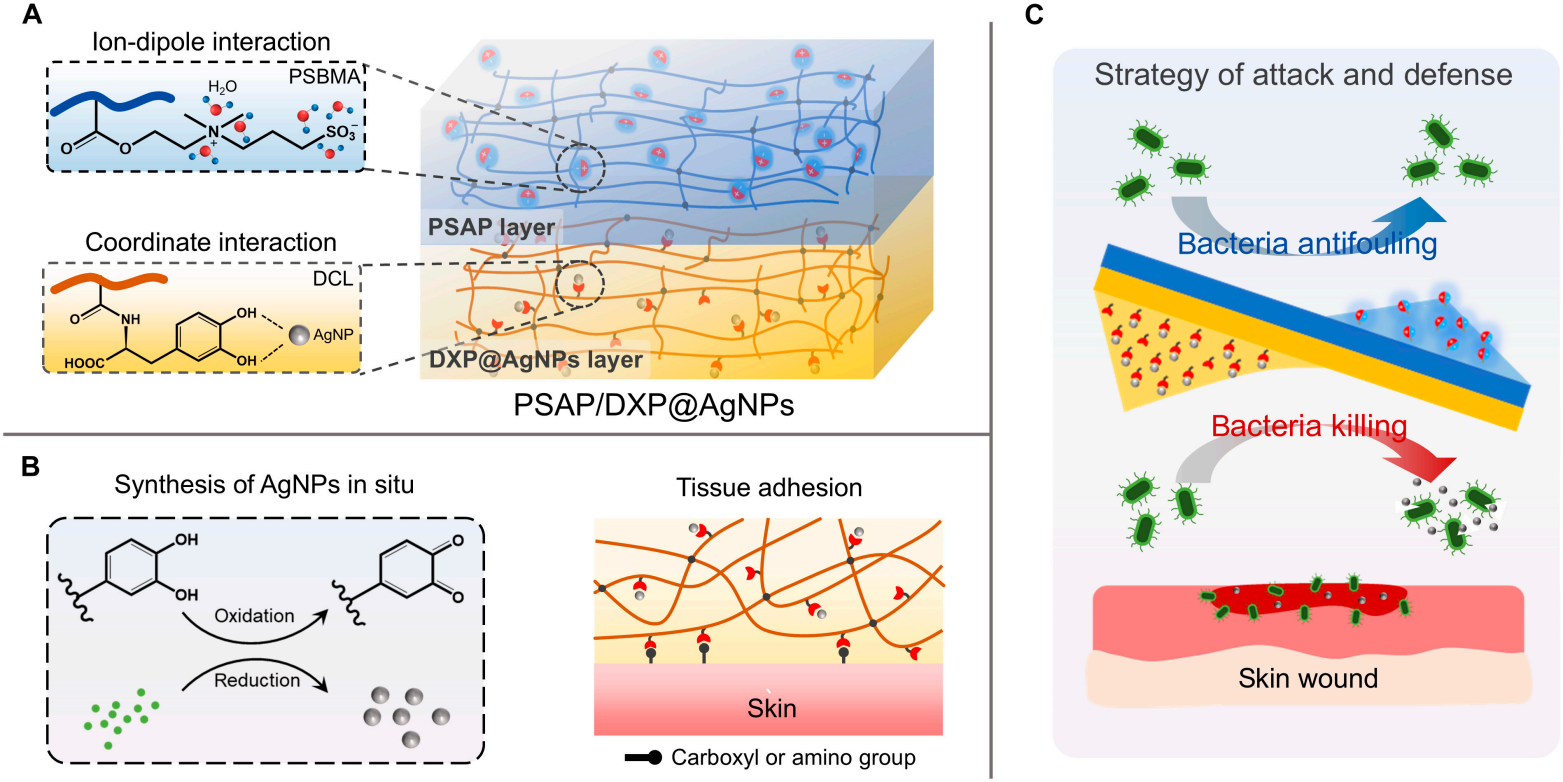
Janus adhesive PSAP/DXP@AgNPs with integrated attack and defense for simultaneously killing bacteria and preventing bacterial contamination. (A) Composition and structure of PSAP/DXP@AgNPs. (B) Synthesis of AgNPs in situ and tissue adhesion of PSAP/DXP@AgNPs. (C) Strategy of attack and defense for bacteria.

Here, a Janus adhesive dual-layer hydrogel (named PSAP/DXP@AgNPs) with integrated attack and defense is successfully proposed to resist bacterial infection for skin wound as shown in Fig. 1. Therein, the well-dispersed AgNPs reduced in situ by the catechol groups in the DXP@AgNPs layer of PSAP/DXP@AgNPs are advantageous to eradicate the existing bacteria on wound, and the hydration effect of zwitterionic PSBMA components in the PSAP layer of PSAP/DXP@AgNPs simultaneously prevents the contamination of various bacteria from external environment. Besides, the asymmetric adhesion of PSAP/DXP@AgNPs ensures its secure fixation at the injured site without the need for medical tape. To evaluate the applicability of PSAP/DXP@AgNPs as hydrogel dressing for the treatment of infected skin wound, the composition/structure, mechanical property, adhesion, swelling, surface antifouling, bacteria killing, and biocompatibility were strictly investigated. In conclusion, PSAP/DXP@AgNPs hydrogel dressing shows promising potential in achieving long-lasting antibacterial property for skin wound, preventing secondary infection in the clinic.

## Results and Discussion

### The composition and structure of PSAP/DXP@AgNPs

**Fig. 2.**
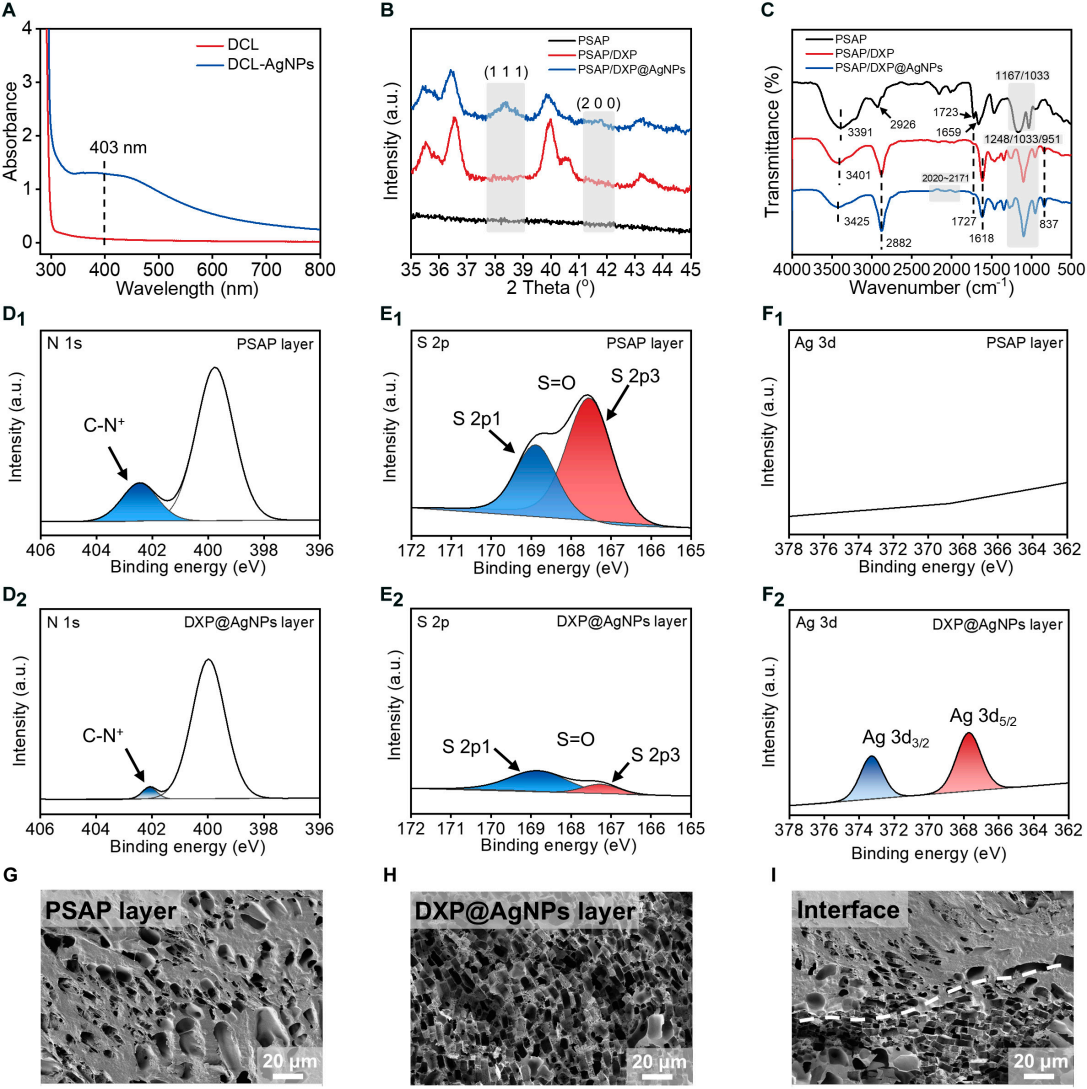
(A) Absorbances of DCL and DCL-AgNPs at 403 nm. (B) XRD spectra of PSAP, PSAP/DXP, and PSAP/DXP@AgNPs. (C) FTIR spectra of PSAP, PSAP/DXP, and PSAP/DXP@AgNPs. XPS high-resolution (D_1_ and D_2_) N 1s, (E_1_ and E_2_) S 2p, and (F_1_ and F_2_) Ag 3d spectra of the PSAP and DXP@AgNPs layers for PSAP/DXP@AgNPs. Cross-section SEM photos of the PSAP layer (G), the DXP@AgNPs layer (H), and the interface (I) of PSAP/DXP@AgNPs hydrogel after lyophilization.

The Janus PSAP/DXP@AgNPs hydrogel dressing integrating antifouling layer and adhesive layer is prepared to simultaneously kill existing bacteria and prevent bacterial contamination from external environment. Poly(*N, N*-dimethylacrylamide-co-levodopa acrylamide) (DCL) is copolymerized from *N*,*N*-dimethylacrylamide and levodopa acrylamide monomers. In addition, the AgNP-free dual-layer hydrogel (named PSAP/DXP) and hydrogel (named PSAP) that resembles the PSAP layer of PSAP/DXP@AgNPs are also prepared. The broad-spectrum antibacterial agent AgNPs are synthesized in situ through the redox between silver ions and catechol groups of DCL in the DXP@AgNPs layer of PSAP/DXP@AgNPs. To verify the production of AgNPs reduced from silver ions by DCL, the ultraviolet-visible (UV-vis) spectrophotometer is adapted to detect the absorbance. As shown in Fig. 2A, it can be observed that there is an obvious absorption peak at 403 nm for DCL after incubation with silver ions, indicating the production of AgNPs in situ [31]. Moreover, the results of x-ray diffraction (XRD) show that crystal peaks of PSAP/DXP@AgNPs at 2θ = 38.3° and 44.5° are respectively attributed to the (1 1 1) and (2 0 0) planes compared to PSAP hydrogel and dual-layer PSAP/DXP hydrogel, which confirms the production of AgNPs in situ once again (Fig. 2B) [20]. Besides, the chemical composition of PSAP/DXP@AgNPs with the bilayer structure is characterized by Fourier transform infrared (FTIR) in Fig. 2C. In the spectrum of PSAP, the peaks at 3,391 and 2,926 cm^−1^ are assigned to the O–H stretching vibration of hydroxyl groups (-OH) of water and C–H stretching vibration, respectively. The peak at 1,723 cm^−1^ is attributed to the ester groups (-O=C–O-) of crosslinked PEG diacrylate (PEGDA) and PSBMA. The peak at 1,659 cm^−1^ is attributed to the quaternary ammonium groups (C-N^+^) of PSBMA. Both of the peaks at 1,167 and 1,033 cm^−1^ are attributed to the sulfonate groups (-SO_3_^−^) of PSBMA [29]. In the spectra of PSAP/DXP and PSAP/DXP@AgNPs, the peak at 2,882 cm^−1^ is also assigned to the C–H stretching vibration. The peaks at 1,618 and 837 cm^−1^ are attributed to the amide groups [O=C–N(CH_3_)_2_] of DCL and C–H bending vibration of benzene rings. The ester groups (-O=C–O-) of crosslinked PEGDA and xanthan gum (XG) are related to the peak at 1,727 cm^−1^. The peaks at 1,248, 1,103, and 951 cm^−1^ are attributed to the ether structure (C–O–C) of crosslinked PEGDA [32]. Interestingly, the wavenumber shift of hydroxyl groups (-OH) at 3,425 cm^−1^ mainly involves the coordination of AgNPs compared to PSAP/DXP [33]. Besides, an appearance of broad band between 2,020 and 2,171 cm^−1^ is related to the formation of Ag–O bond [34].

Due to the asymmetric function of PSAP/DXP@AgNPs, the chemical structure of the PSAP layer for PSAP/DXP@AgNPs is different from that of the DXP@AgNPs layer, which is proved by x-ray photoelectron spectroscopy (XPS) (Fig. 2D to F). According to the high-resolution N 1s spectrum in Fig. 2D_1_, the binding energy peaks at 402.4 eV fitted from the N 1s core-level spectrum are accounted for the quaternary ammonium groups (C-N^+^) of PSBMA in the PSAP layer of PSAP/DXP@AgNPs. At the same time, the binding energy peaks at 168.8 and 167.6 eV respectively fitted from S 2p1 and S 2p3 core-level spectra are accounted for the sulfonate groups (-SO_3_^−^) of PSBMA (Fig. 1E_1_). Meanwhile, those characteristic high-resolution spectra for N 1s and S 2p can also be observed in the DXP@AgNPs layer with weak intensity (Fig. 2D_2_ and E_2_). The results indicate that SBMA monomers partly penetrate into the DXP@AgNPs layer through interface during gelation, which is beneficial to construct a reliable interfacial structure. Besides, the high-resolution C 1s spectrum of the DXP@AgNPs layer is different from that of the PSAP layer as shown in Fig. S1. The binding energy peaks at 286.4 eV fitted from C–O core-level spectra appear in both 2 layers. Due to the many PEG segments in the DXP@AgNPs layer, the relative intensity of C–O core-level spectrum for the DXP@AgNPs layer is stronger than that for the PSAP layer, which is in accordance with the Janus structure of PSAP/DXP@AgNPs hydrogel. As shown in Fig. 2F_1_ and F_2_, the binding energy peaks at 373.2 and 367.7 eV respectively fitted from Ag 3d_3/2_ and Ag 3d_5/2_ core-level spectra just appear in the DXP@AgNPs layer without appearance in the PSAP layer, indicating that antibacterial AgNPs just distribute in the DXP@AgNPs layer.

As the asymmetric chemical composition exists in PSAP/DXP@AgNPs demonstrated by XPS, the asymmetric structure of PSAP/DXP@AgNPs should be observed by scanning electron microscopy (SEM). As shown in Fig. 2G, the cross-section morphology of the PSAP layer for PSAP/DXP@Ag shows 3-dimensional porous network with low pore density and thick pore wall, which is attributed to the electrostatic interactions between quaternary ammonium and sulfonate groups of PSBMA [35]. Meanwhile, the DXP@AgNPs layer of PSAP/DXP@AgNPs shows a loose and porous network structure observed from the cross-section morphology in Fig. 2H. Between the 2 layers, the tight combination of the interfacial structure exists without any detachment (Fig. 2I). It can be reasonably supposed that the asymmetric structure of PSAP/DXP@AgNPs has the potential to provide a barrier effect to block the bacterial invasion relying on the dense PSAP layer and release AgNPs to kill existing bacteria relying on the loose DXP@AgNPs layer.

### The mechanical property, thermal stability, and adhesion of PSAP/DXP@AgNPs

**Fig. 3.**
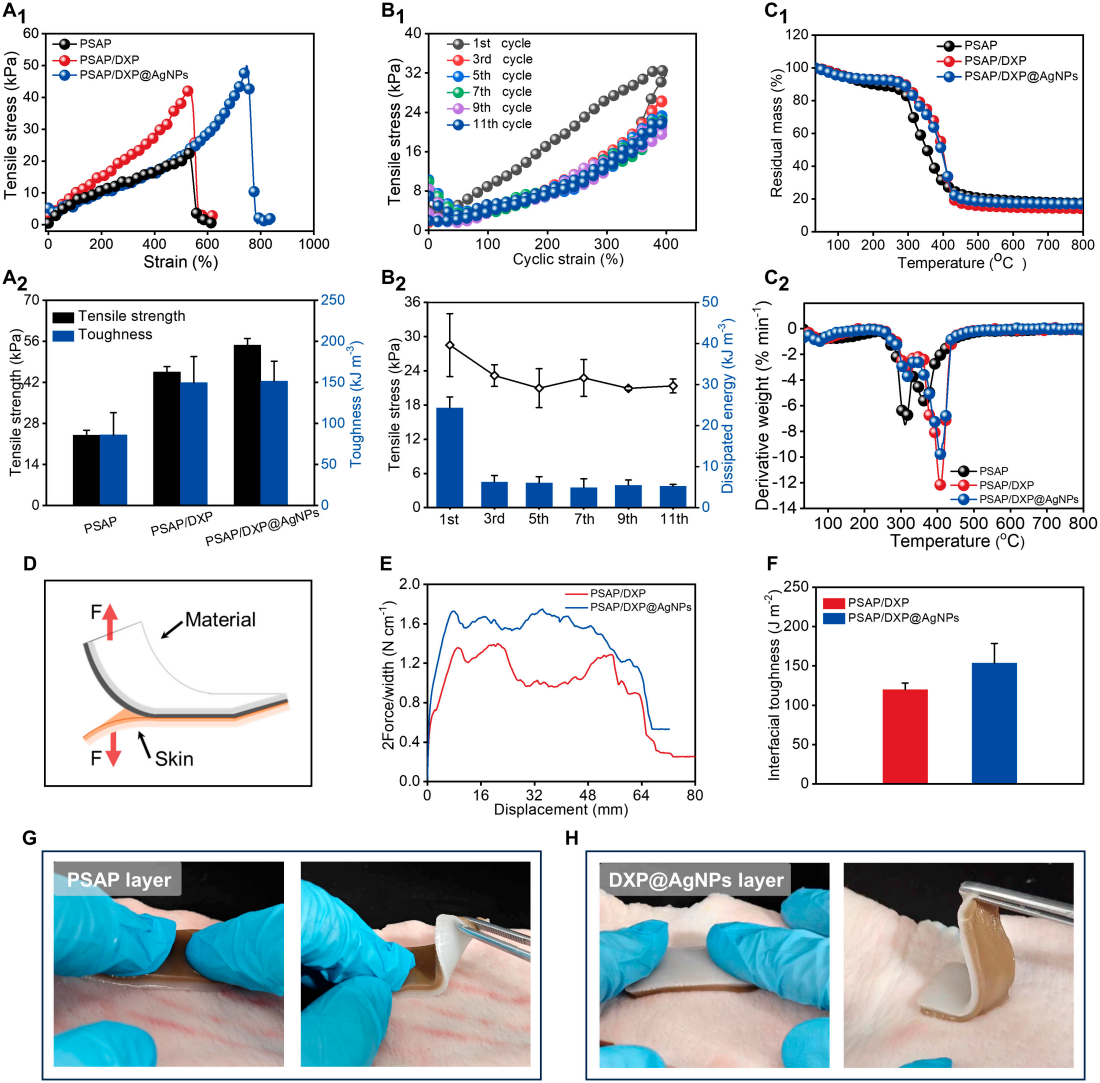
(A_1_) Tensile stress–strain curves of PSAP, PSAP/DXP, and PSAP/DXP@AgNPs hydrogels and (A_2_) relevant tensile strength and toughness values. (B_1_) Eleven cyclic tensile stress–strain curves of PSAP/DXP@AgNPs and (B_2_) relevant tensile stress and dissipated energy values. (C_1_) TGA and (C_2_) DTGA curves of PSAP, PSAP/DXP, and PSAP/DXP@AgNPs. (D) Scheme of 180° peeling experiments of materials. (E) The 180° peeling curves of PSAP/DXP and PSAP/DXP@AgNPs and (F) relevant interfacial toughness values. Adhesions of (G) PSAP and (H) DXP@AgNPs layers of PSAP/DXP@AgNPs toward skin tissues.

The dynamic characteristic of skin requires wound dressing to be stretchable, complying with the shape change of skin under force. So, the tensile experiments are conducted to study the tensile property of PSAP/DXP@AgNPs. As shown in Fig. 3A_1_ and A_2_, the tensile stress–strain result shows that the strain of PSAP reaches close to 600% with a tensile strength of 24 kPa, resulting in a tensile toughness of 86.09 kJ m^−3^. PSAP/DXP shows a tensile strength of 45.56 kPa and a tensile toughness of 149.8 kJ m^−3^ with similar strain, which is enhanced by the dense PSAP layer. The tensile property of PSAP/DXP@AgNPs is 54.72 kPa with a strain of 800%, resulting in a tensile toughness of 151.6 kJ m^−3^. It is mainly due to the coordination between AgNPs and ligand groups including carboxyl and catechol groups, which increases the physical crosslinked sites in PSAP/DXP@AgNPs. Besides, as shown in Fig. 3B_1_ and B_2_, all 11 successive loading–unloading tensile cycles under 400% strain exhibit almost unchanged tensile strengths and dissipated energies except for the first cycle. It is found that the hysteresis loops from the 2nd to 11th cycles are nearly overlapped and less than that of the 1st cycle, implying that PSAP/DXP@AgNPs suffers from structural changes during the 1st tensile cycle and retains the same network structure in the following cycles [36]. The results indicate that PSAP/DXP@AgNPs as wound dressing has good fatigue resistance and stretchability, which can adapt to the shape change of skin when used.

In order to investigate the influence of AgNPs on thermal stability, the thermogravimetric analysis (TGA) and derivative TGA (DTGA) are carried out. As shown in Fig. 3C_1_, in the first degradation stage of PSAP, PSAP/DXP, and PSAP/DXP@AgNPs (35 to 208 °C, 35 to 143 °C, and 35 to 160 °C), the weight losses of PSAP, PSAP/DXP, and PSAP/DXP@AgNPs respectively occupy 11%, 5%, and 6%, due to the evaporation of moisture. With the increase of temperature (270 to 366 °C, 266 to 468 °C, and 268 to 469 °C), the weight losses of 65.71%, 73.51%, and 70.63% are attributed to the degradation of polymer for PSAP, PSAP/DXP, and PSAP/DXP@AgNPs, respectively, releasing volatiles such as moisture and CO_2_. In the third degradation stage of PSAP, PSAP/DXP, and PSAP/DXP@AgNPs (462 to 800 °C, 468 to 800 °C, and 469 to 800 °C), the weight losses of PSAP, PSAP/DXP, and PSAP/DXP@AgNPs occupy 3.72%, 3.23%, and 2.52% with the carbonization process, resulting in a total residual weight of 17.84%, 14.43%, and 17.49%, respectively. The results indicate that the initial degradable temperatures or final degradable temperatures for PSAP, PSAP/DXP, and PSAP/DXP@AgNPs are similar. The thermal degradation can be further analyzed by DTGA. As shown in Fig. 3C_2_, the maximum degradation rate of PSAP/DXP is 3.16% min^−1^, close to that of 7.32% min^−1^ for PSAP/DXP. The maximum degradation rate of PSAP is 5.63% min^−1^ at 362.6 °C, lower than that of 12.1% min^−1^ at 407.7 °C for PSAP/DXP due to the strong electrostatic interaction induced by PSBMA. Compared to PSAP/DXP, PSAP/DXP@AgNPs shows similar maximum degradation rate at 311.78 °C, but a lower maximum degradation rate (9.66% min^−1^) at 407.7 °C. The results indicate that PSAP/DXP@AgNPs exhibits good thermal stability that is originated from the contributions of electrostatic interaction in the PSAP layer and stable inorganic AgNPs in the DXP@AgNPs layer.

The adhesive property of PSAP/DXP@AgNPs is helpful to be fitted at skin wound tightly without additional assistance of medical tape. Hence, the 180° peeling experiments are conducted to evaluate the tissue adhesion of PSAP/DXP@AgNPs (Fig. 3D). As shown in Fig. 3E, the adhesive strength first increases and then maintains a plateau region until complete peeling of sample from porcine skin with the tensile process, which is suitable for adhesion descriptions of PSAP/DXP and PSAP/DXP@AgNPs. The interfacial toughness of PSAP/DXP is 119.88 J m^−2^ mediated by interactions of the catechol group and amidation, whereas PSAP/DXP@AgNPs exhibits a notably higher interfacial toughness of 153.38 J m^−2^ than PSAP/DXP (Fig. 3F). The improvement of adhesion is attributed to the inner AgNPs that crosslink the network by coordination including Ag–catechol group interaction and Ag–carboxyl group interaction, increasing the energy dissipation [37,38]. Besides, asymmetric adhesion of PSAP/DXP@AgNPs is described in Fig. 3G and H. After the PSAP layer of PSAP/DXP@AgNPs is pressed on porcine skin for a while, PSAP/DXP@AgNPs is easily moved from the skin tissue, exhibiting antiadhesive phenomenon. When the DXP@AgNPs layer of PSAP/DXP@AgNPs contacts the surface of porcine skin, the adhesive strap is successfully observed from the peeling interface, exhibiting obvious adhesion. Therefore, the asymmetric adhesion of PSAP/DXP@AgNPs dressing is helpful to ensure the complete fit between the material and wound, avoiding the inconvenient fixation of medical tape.

### Bacteria-killing property of PSAP/DXP@AgNPs

**Fig. 4.**
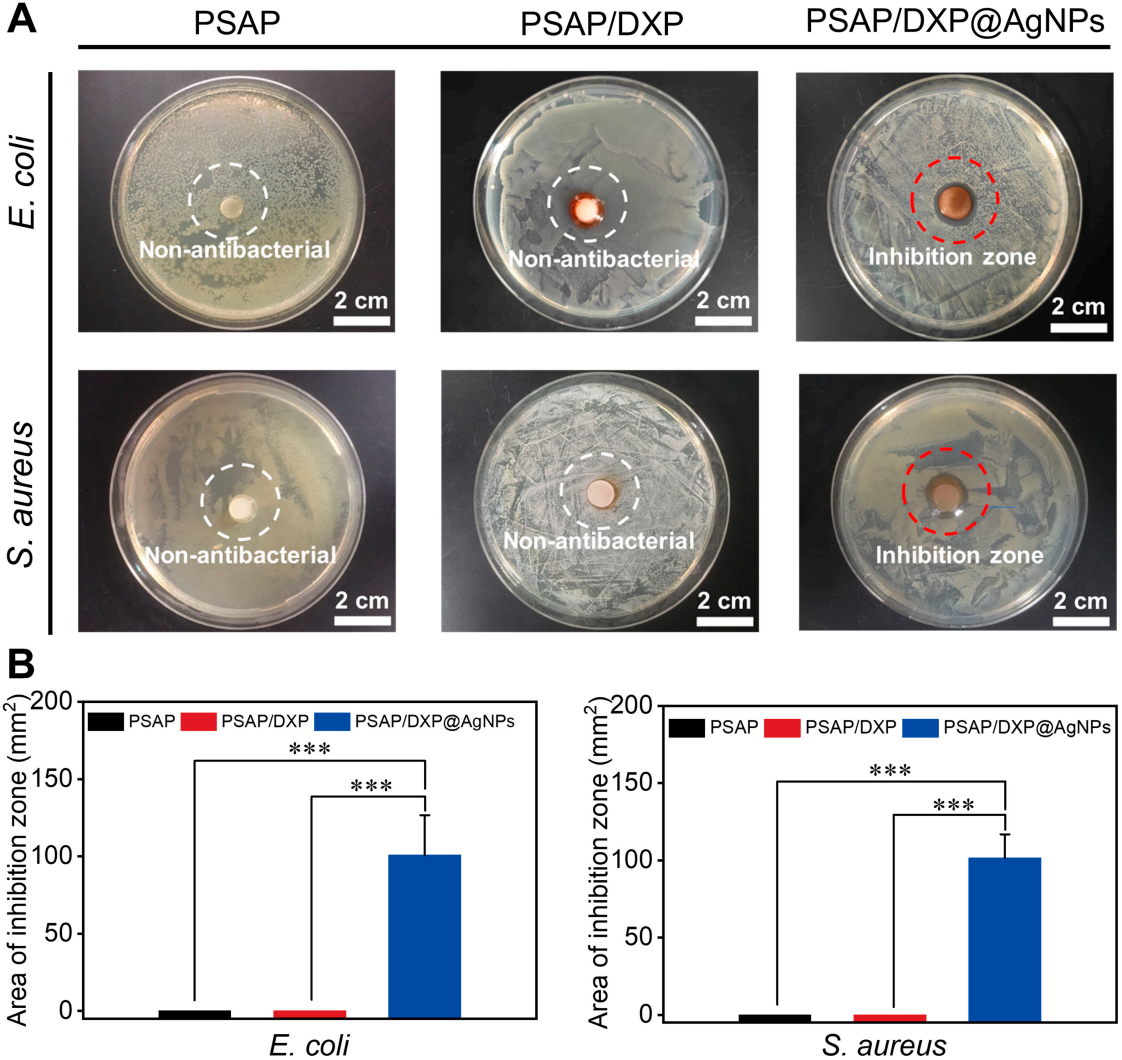
(A) Zone of inhibition produced by PSAP, PSAP/DXP, and PSAP/DXP@AgNPs for *E. coli* and *S. aureus* and relevant (B) areas of inhibition zone. *P* values are determined by one-way analysis of variance (ANOVA) with Tukey’s multiple comparisons test. ***P < 0.001 indicates statistical difference between different groups.

PSAP/DXP@AgNPs with integrated attack and defense is designed to resist bacterial infection for skin wound. The DXP@AgNPs layer of PSAP/DXP@AgNPs is capable of killing the existing bacteria on infected wound, impeding the further deterioration of wound. A preliminary evaluation of the antibacterial properties of PSAP/DXP@AgNPs by testing the inhibition zone for *Escherichia coli* or *Staphylococcus aureus* is required. PSAP, PSAP/DXP, and PSAP/DXP@AgNPs are placed on the solid culture media covered by bacterial solution and then coincubated for 24 h to measure the size of inhibition zone. As shown in Fig. 4A, both the PSAP and PSAP/DXP groups show almost negligible sizes of inhibition zone, indicating minimal antibacterial effects against *E. coli* and *S. aureus*. In contrast, after coincubation with both bacterial strains, a clear inhibition zone is observed around PSAP/DXP@AgNPs (101.64 mm^2^ for *E. coli* and 100.78 mm^2^ for *S. aureus*) (Fig. 4B). The results indicate that broad-spectrum antibacterial AgNPs are gradually released from the DXP@AgNPs layer of PSAP/DXP@AgNPs, spreading outward from the center, inhibiting bacterial proliferation, and ultimately forming observable inhibition zone.

**Fig. 5.**
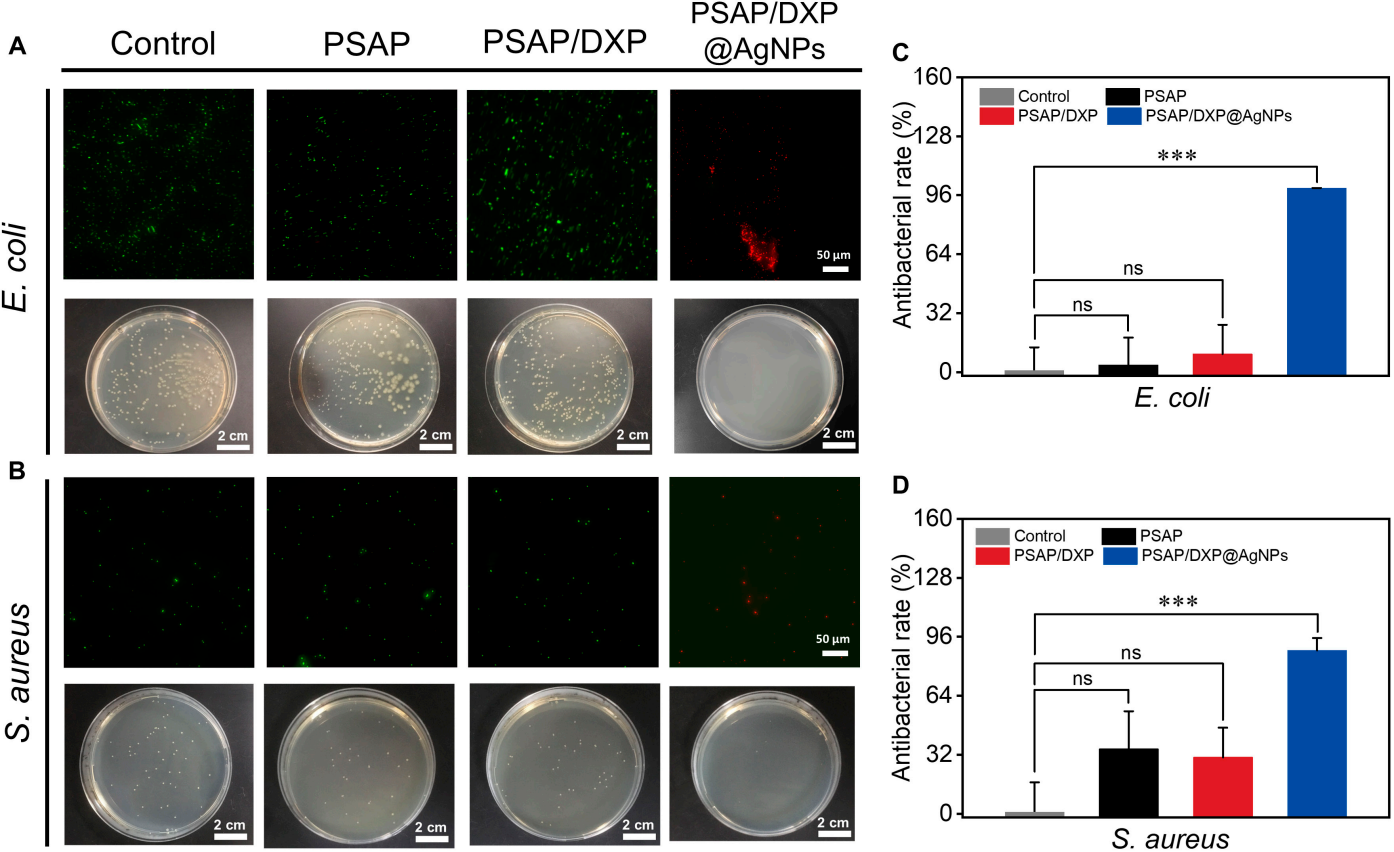
Antibacterial effects (live/dead staining and bacterial count) of PSAP, PSAP/DXP, and PSAP/DXP@AgNPs against (A) *E. coli* and (B) *S. aureus* and relevant antibacterial rates of (C) *E. coli* and (D) *S. aureus*. *P* values are determined by one-way ANOVA with Tukey’s multiple comparisons test. ***P < 0.001 indicates statistical difference between different groups. ns, no significance (*P* > 0.05).

In order to further analyze the antibacterial properties, bacteria are coincubated with PSAP, PSAP/DXP, and PSAP/DXP@AgNPs for 2 h at 37 °C. Subsequently, the diluted bacteria added on the plate are incubated for 12 h to calculate the bacterial count, thereby evaluating the antibacterial property. As shown in Fig. 5A and B, a large number of *E. coli* colonies proliferate on the solid culture media for control, PSAP, and PSAP/DXP groups. The live/dead staining of *E. coli* demonstrates that almost all bacteria are alive (green staining) for control, PSAP, and PSAP/DXP groups. In contrast, there are few *E. coli* colonies on solid culture media for the PSAP/DXP@AgNPs group, and almost all *E. coli* are observed as dead (red staining), exhibiting efficient antibacterial rate of 99%. As shown in Fig. 5C and D, both the PSAP and PSAP/DXP groups also exhibit undesirable antibacterial rates for *S. aureus* with many living bacteria (green staining). PSAP/DXP@AgNPs is able to kill *S. aureus* (red staining), exhibiting a higher antibacterial rate of 88%. In brief, PSAP/DXP@AgNPs shows promising potential for eradicating infected bacteria on skin wound.

### Bacteria-antifouling property of PSAP/DXP@AgNPs

**Fig. 6.**
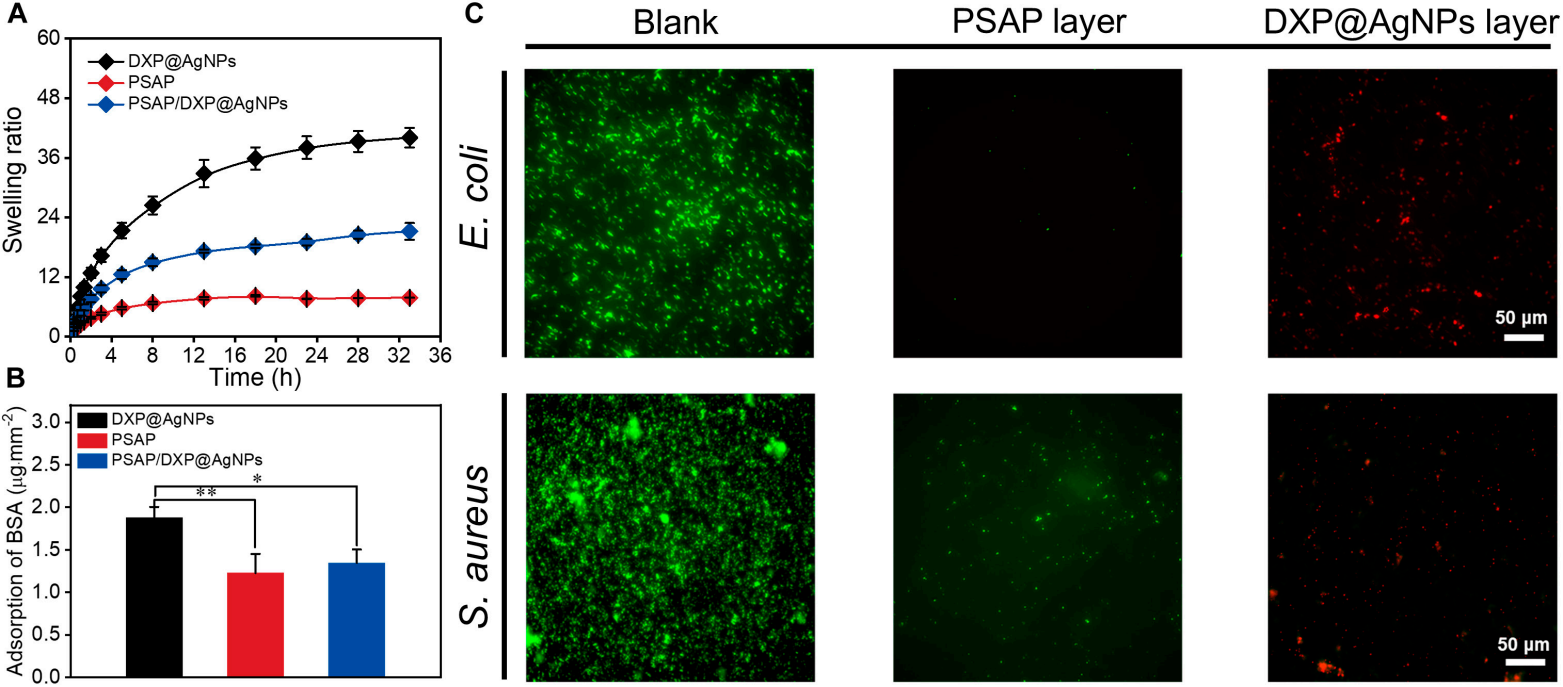
(A) Swelling ratios of DXP@AgNPs, PSAP, and PSAP/DXP@AgNPs groups. (B) BSA adsorption capacities of DXP@AgNPs, PSAP, and PSAP/DXP@AgNPs groups. (C) Live/dead staining of *E. coli* and *S. aureus* cocultured on blank plate, PSAP layer, and DXP@AgNPs layer of PSAP/DXP@AgNPs. *P* values are determined by one-way ANOVA with Tukey’s multiple comparisons test. *P < 0.05 and **P < 0.01 indicate statistical difference between different groups.

PSAP/DXP@AgNPs with integrated attack and defense can not only kill the existing bacteria on skin wound but also prevent the contamination of bacteria from external environment relying on the PSAP layer of PSAP/DXP@AgNPs. The hydration effect of zwitterionic PSBMA components in the PSAP layer enables to resist the anchoring of adhesive proteins of bacteria and decrease the bacterial accumulation, lowering the risk of secondary infection. Hence, the commonly used bovine serum albumin (BSA) is chosen as the model protein to study the protein-antifouling property of PSAP/DXP@AgNPs. The antifouling properties of PSAP and hydrogel (named DXP@AgNPs) that resembles the DXP@AgNPs layer of PSAP/DXP@AgNPs are also tested compared with that of PSAP/DXP@AgNPs. The equilibrium swelling states of DXP@AgNPs, PSAP, and PSAP/DXP@AgNPs groups are tested (equilibrium swelling ratios: 39.1, 7.35, and 19.2) (Fig. 6A). To quantitatively determine the adsorption capacity of BSA for materials, the standard bicinchoninic acid (BCA) method is adopted. Calculated from the standard curve, the adsorption capacities of DXP@AgNPs, PSAP, and PSAP/DXP@AgNPs are 1.88, 1.23, and 1.35 μg mm^−2^, respectively (Fig. 6B). This is due to the fact that the synergistic effect of hydrated shell on the PSAP surface and the dense network structure in the inner PSAP, both involving zwitterionic PSBMA, limits the adsorption of BSA, compared to DXP@AgNPs. Due to the favorable protein-antifouling property of PSBMA in the PSAP layer of PSAP/DXP@AgNPs, the BSA adsorption of PSAP/DXP@AgNPs is obviously restricted. Afterward, bacteria-antifouling experiments are needed to assess the bacteria-antifouling property of PSAP/DXP@AgNPs. As shown in Fig. 6C, after incubation in a plate well for 24 h (blank group), the live/dead staining of *E. coli* and *S. aureus* presents green fluorescence, indicating that a lot of living bacteria exist. In contrast, the live/dead staining of *E. coli* and *S. aureus* cocultured on the PSAP layer of PSAP/DXP@AgNPs presents noticeably less green fluorescence, indicating an excellent bacteria-antifouling property. The results exhibit that the PSAP layer of PSAP/DXP@AgNPs containing zwitterionic PSBMA components resists the anchoring of adhesive proteins of bacteria, decreasing the bacterial accumulation. Besides, the live/dead staining of *E. coli* and *S. aureus* cocultured on the DXP@AgNPs layer of PSAP/DXP@AgNPs presents sparse red fluorescence, indicating that released AgNPs kill the bacteria and restrict further proliferation.

### Biocompatibility of PSAP/DXP@AgNPs

**Fig. 7.**
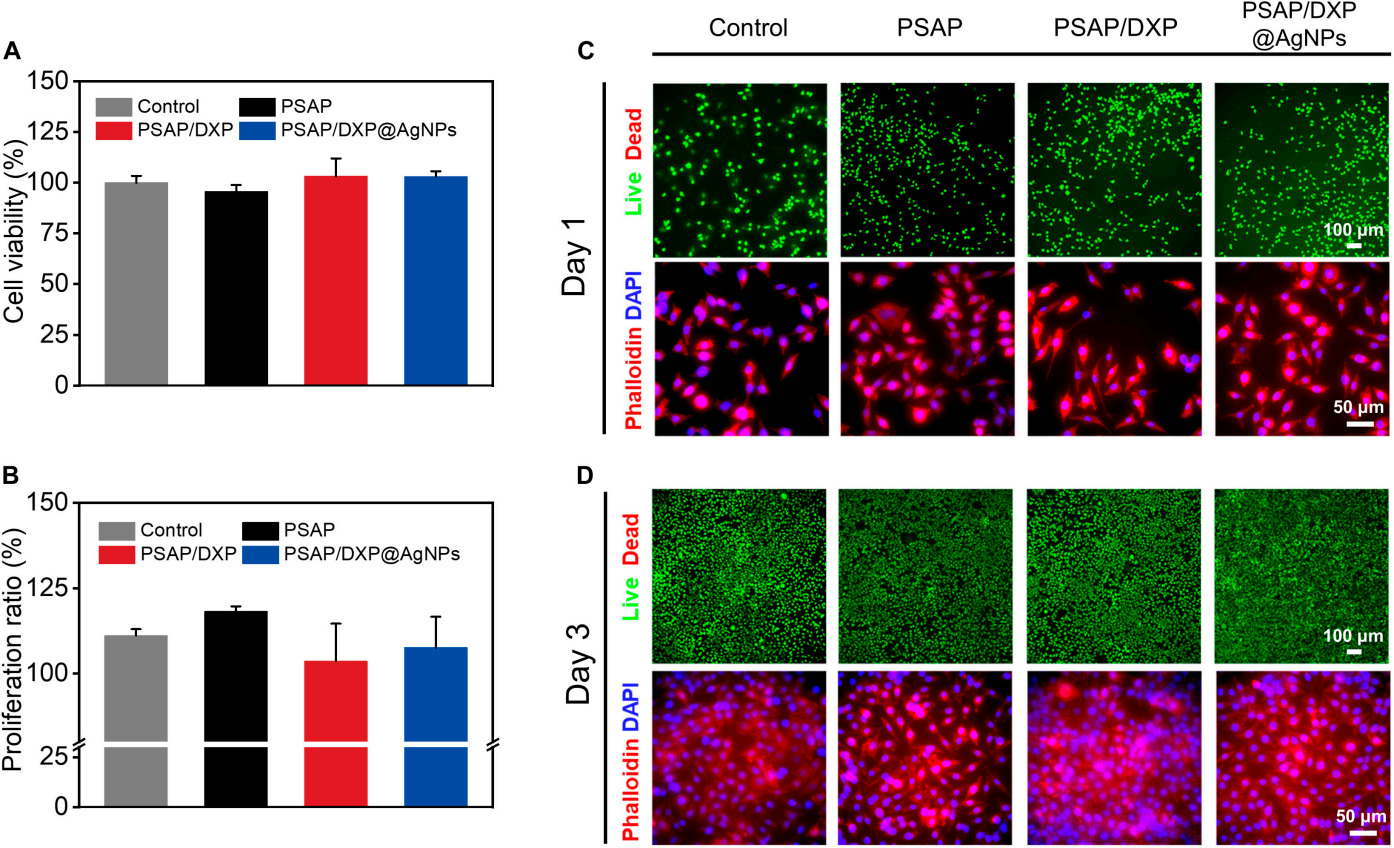
(A) L929 cell viabilities after coculture with extracts of control, PSAP, PSAP/DXP, and PSAP/DXP@AgNPs groups for 1 day. (B) Proliferation ratios of control, PSAP, PSAP/DXP, and PSAP/DXP@AgNPs groups for 3 days. (C) Live/dead staining and morphological staining of L929 cells after coculture with extracts of PSAP, PSAP/DXP, and PSAP/DXP@AgNPs for 1 day and (D) live/dead and morphological staining for 3 days.

Biocompatibility is a vital indicator for biomaterials, which can be evaluated by cytotoxicity. L929 cells are respectively cocultured with different samples (PSAP, PSAP/DXP, and PSAP/DXP@AgNPs) to assess the cytotoxicity. The cell viability is quantitatively determined using CCK-8 assay. The cell viabilities of all groups exceed 90% for 1 day, higher than 75%, indicating low cytotoxicity (Fig. 7A). Then, L929 cells are continued to be cocultured with PSAP, PSAP/DXP, and PSAP/DXP@AgNPs samples for 3 days, respectively. The results show that L929 cells present certain proliferation compared to the cells cocultured for 1 day (Fig. 7B). As shown in Fig. 7C, the live/dead staining of L929 cells for all groups present green fluorescence without red fluorescence, showing that cells remained alive, consistent with the CCK-8 assay results. Additionally, the morphological staining results show L929 cells with round cell nuclei (blue) and spreading cytoskeleton (red), indicating good cellular adhesion to plate. After the L929 cells are cultured for 3 days, the live/dead staining and morphological staining of cells are shown in Fig. 7D. The live/dead staining of L929 cells present more intensive green fluorescence, indicating the cellular proliferation. Due to the gradual reduction of growth space in well with the proliferation, the round and intact nuclei surrounded by the partly overlapped cytoskeleton is observed by morphological staining. In conclusion, the low cytotoxicity of PSAP/DXP@AgNPs reveals excellent biocompatibility.

## Conclusion

In summary, we designed a Janus PSAP/DXP@AgNPs hydrogel with integrated attack and defense, which has the potential to simultaneously kill the existing bacteria on wound and prevent the contamination of bacteria from external environment. To characterize the material and evaluate the related properties in detail, a series of experiments are conducted. Due to the different chemical compositions between the 2 layers, PSAP/DXP@AgNPs with 3-dimensional porous network shows low pore density in the PSAP layer and loose structure in the DXP@AgNPs layer. The tensile experiments demonstrate that PSAP/DXP@AgNPs possesses good fatigue resistance and stretchability, which is useful to adapt dynamic shape change of skin. The asymmetric adhesion of PSAP/DXP@AgNPs with an interfacial toughness of 153.38 J m^−2^ toward skin, relying on the DXP@AgNPs layer, is meaningful to ensure the complete fit of hydrogel dressing onto wound without the need for medical tape. Besides, the catechol groups dangling on DCL enable to reduce silver ions to AgNPs in situ, leading to well-dispersed AgNPs in the DXP@AgNPs layer of PSAP/DXP@AgNPs. The AgNPs released from the DXP@AgNPs layer of PSAP/DXP@AgNPs restrict the bacterial proliferation with obvious zone of inhibition for *E. coli* and *S. aureus* (101.64 mm^2^ for *E. coli* and 100.78 mm^2^ for *S. aureus*), exhibiting good antibacterial rates toward those bacterial strains of 99% and 88%, respectively. Meanwhile, the hydrated PSAP layer of PSAP/DXP@AgNPs containing zwitterionic PSBMA components is able to prevent bacterial contamination by resisting the approach of adhesive protein of bacteria. Moreover, the cell experiment demonstrates that L929 cells enable to maintain cellular viability and healthy spreading cytoskeleton. In conclusion, the asymmetrically adhesive PSAP/DXP@AgNPs hydrogel with integrated attack and defense simultaneously achieves bacteria-killing and bacteria-antifouling properties, which makes it a promising candidate for use as a novel wound dressing in the clinic.

## Materials and Methods

### Main reagents

XG (number-average molecular weight = 8.08 × 10^6^ Da, USP), levodopa (99%), acryloyl chloride (96%), azodiisobutyronitrile (AIBN; recrystallization 99%), Irgacure 2959 (I_2959_, 98%), *N*-hydroxy succinimide (NHS; 98%), *N*,*N*-dimethylacrylamide (DMA; >99.5%), *N*,*N*′-methylenebis (2-propenamide) (MBA; 99%), and triethylamine (TEA; 99%) were purchased from Aladdin Biochemical Technology Co. Ltd. (Shanghai, China). Acrylamide (AM; 99%), *N*-(3-dimethylaminopropyl)-*N*′-ethylcarbodiimide hydrochloride (EDC^**.**^HCl; 98.5%), dimethyl sulfoxide (DMSO; 98%), ethylacetate (EA; 99%), *n*-hexane (97%), tetrahydrofuran (THF; 99%), and dichloromethane (CH_2_Cl_2_; 99%) were purchased form Macklin Biochemical Co. Ltd. (Shanghai, China). NaCl (99.8%), Na_2_B_4_O_7_^**.**^10H_2_O (99.5%), Na_2_CO_3_ (99%), K_2_CO_3_ (99%), MgSO_4_ (99%), and PEG (35,000 Da) were purchased from Sigma-Aldrich (Shanghai, China). Sulfobetaine methacrylamide (SBMA; 98%) was purchased from Yuanye Bio-Technology Co. Ltd. (Shanghai, China). AgNO_3_ (99.8%) was purchased from Xilong Scientific Co. Ltd. (Shantou, China). Luria–Bertani (LB) broth was purchased from Hope Bio-Technology Co. Ltd. (Qingdao, China). BSA and agar were purchased from Biofroxx (Germany). *E. coli* (ATCC 25922) and *S. aureus* (ATCC 6538) were purchased from Huankai Microbial Sci. & Tech. Co. Ltd. (Guangzhou, China). SYTO 9-PI Live and Dead Bacteria Stain Kit was purchased from Invitrogen (USA). Cell counting kit-8 (CCK-8) was purchased from MedChemExpress (New Jersey, USA). α-Modified Eagle’s medium (α-MEM), fetal bovine serum (FBS), and penicillin–streptomycin solution were purchased from Bosco Biotechnology Co. Ltd. (Shanghai, China). Propidium iodide (PI), rhodamine-labeled phalloidin, fluorescein diacetate (FDA), and 4′,6-diamidino-2-phenylindole (DAPI) were purchased from Solarbio Science & Technology Co. Ltd. (Beijing, China). All chemical reagents were used as received without any further purification unless otherwise stated.

### Synthesis of PEGDA, levodopaMA, and DCL

The specific procedures of synthesis for PEGDA, levadopaMA, DCL, and fluorescein-labeled DCL were described in detail in the Supplementary Materials. The proton nuclear magnetic resonance (^1^H NMR) spectra of PEGDA, levodopaMA, and DCL are shown in Figs. S2 to S4.

### The preparation of PSAP/DXP@AgNPs hydrogel

The dual-layer structure of PSAP/DXP@AgNPs hydrogel is formed by constructing the PSAP layer on the surface of the DXP@AgNPs layer. First, 190 mg of DCL was dissolved in 1.6 ml of 0.625 mg ml^−1^ AgNO_3_ solution and stirred for 3 days to synthesize AgNPs in situ in the dark. The mixture of 2 mg of XG, 2 mg of I_2959_, and 190 mg of PEGDA was added into solution above to form A pregel solution. The A pregel solution was polymerized to form the DXP@AgNPs layer of PSAP/DXP@AgNPs in rectangle Teflon mold under an irradiation of 365 nm UV light for 15 min. Then, the mixture of 5 mg of I_2959_, 288 mg of SBMA,120 mg of AM, 72 mg of PEGDA, and 1 mg of *N*,*N*-methylene bisacrylamide (MBAA) was dissolved in 1.6 ml of deionized water to form B pregel solution. The B solution was poured onto the DXP@AgNPs layer in mold and polymerized to form the PSAP layer on the DXP@AgNPs layer by irradiation of 365-nm UV light for 20 min, preparing dual-layer PSAP/DXP@AgNPs hydrogel. Additionally, the AgNP-free dual-layer hydrogel (PSAP/DXP) was prepared by the similar method without in situ reduction of silver ions. The PSAP hydrogel was prepared by the UV light-initiated gelation of B pregel solution alone. The DXP@AgNPs hydrogel was prepared by the gelation of A pregel solution.

### Characterizations

The samples (PEGDA, levodopaMA, and DCL copolymer) were dissolved in D_2_O, and the chemical structures were characterized by ^1^H NMR. UV-vis absorption spectra of DCL and DCL-AgNPs were detected by a spectrophotometer (UV-2550, Shimadzu, Japan) with a resolution of 1 nm by using a 1-cm path length quartz cuvettes. PSAP, PSAP/DXP, and PSAP/DXP@AgNPs were performed using an FTIR spectroscope (Nicolet iS50, Thermo Fisher Scientific, USA) with a resolution of 0.4 cm^−1^ and a test range of 4,000 to 500 cm^−1^. XPS (Thermo Kalpha, Thermo Fisher Scientific, USA) was used to test N, S, and Ag elements of PSAP/DXP@AgNPs with mono-Al Kα radiation. The crystal structures of PSAP, PSAP/DXP, and PSAP/DXP@AgNPs were characterized by x-ray diffractometer with a scanning rate of 5° min^−1^ and a range of 5 to 80°. TGA and DTGA of PSAP, PSAP/DXP, and PSAP/DXP@AgNPs were carried out with TA Instruments (TG209F1, NETZSCH, Germany) in N_2_ atmosphere at a heating rate of 10 °C min^−1^ from 35 to 800 °C. The cross-section morphology of PSAP/DXP@AgNPs was observed by SEM (Gemini 300, Carl Zeiss, Germany) under an acceleration voltage of 3.0 kV.

### Tensile mechanical property

The mechanical properties of hydrogel samples (PSAP, PSAP/DXP, and PSAP/DXP@AgNPs) were measured by universal mechanical testing instrument (HZ-1004B, Hengzhun Instrument Technology Co. Ltd., China) with a 20-kg load cell at ambient temperature. The dumbbell-shaped hydrogel sample (width: 2 mm, thickness: 1 mm, gauge length: 15 mm) was clamped on the fixture and then used to measure the uniaxial tensile property at a tensile rate of 100 mm min^−1^ until the sample was fractured. The maximum tensile strength of hydrogel sample was determined at the fractured strain, and the toughness could be determined by calculating the integral area of the tensile stress over the strain until fracture. For the cyclic tensile measurement, the dumbbell-shaped sample was stretched to preset strain and then returned to its original length. The rate of loading–unloading process kept unchanged at 100 mg ml^−1^. The maximum tensile stress of the hydrogel sample was determined at the preset maximum strain, and the dissipated energy could be determined by calculating the integral loop area of the loading–unloading curve.

### Adhesive property

The 180° peeling experiment was conducted on a texture analyzer (TA.XTC-20, Bosin Tech, China) to determine the adhesive property of asymmetric DJP barrier toward fresh porcine skin. The samples (PSAP/DXP and PSAP/DXP@AgNPs) were pressed against porcine skins and mediated by EDC/NHS solution (12 mg ml^−1^) at ambient temperature to form adhesions. The force–displacement curves were obtained from the 180° peeling experiments at a rate of 100 mm min^−1^. The interfacial toughness value of adhesion (Γ) could be calculated from Eq. 1:

$$\Gamma =\frac{2F_{\text{plateau}}}{W}$$ (1)

where *F*_plateau_ represents the plateau force during the peeling process and *W* represents the width of the sample.

### Swelling ratio

The samples (PSAP, DXP@AgNPs, and PSAP/DXP@AgNPs) were dried in air. Then, the dried samples were immersed in phosphate-buffered saline (PBS) for different time at ambient temperature. The swelling ratio was calculated from the mass of dried and swollen sample according to Eq. 2:

$$\text{Swelling ratio}=\frac{W_{t}-W_{0}}{W_{0}}$$ (2)

where *W*_t_ represents the mass after immersion for *t* and *W*_0_ represents the initial dry mass.

### Bacteria-killing property

To preliminary evaluate the antibacterial effects of samples (PSAP, PSAP/DXP, and PSAP/DXP@AgNPs) against *E. coli* and *S. aureus*, the experiment of inhibition zone was conducted as follows. One hundred microliters of 10^6^ colony-forming units (CFU) ml^−1^ bacterial suspension was added on the LB-agar plate. Then, the bacteria on LB-agar plate were covered by the sample of hydrogel disc and cocultured for 24 h at 37 °C. Area of inhibition zone could be calculated from Eq. 3:

$$\text{Area of inhibition zone}\;\left({\mathrm{mm}}^{2}\right)=\pi \times \left(R^{2}-r^{2}\right)$$ (3)

where *R* represents the radius of the outer circle and *r* represents the radius of the inner circle.

The bacteria-killing properties of samples (PSAP, PSAP/DXP, and PSAP/DXP@AgNPs) against *E. coli* and *S. aureus* by the contact method were evaluated, which refers to the Tian’s protocol [39]. The sample was placed onto the wells of a 6-well plate and sterilized by UV radiation. Then, 10 μl of 10^8^ CFU ml^−1^ bacterial suspension was cocultured with the hydrogel sample for 2 h at 37 °C. Subsequently, the bacteria were washed by 2 ml of PBS and diluted 10^4^ times to obtain the final bacterial suspension. The 50-μl diluted bacterial suspension was added on LB-agar plate and cocultured for 14 h at 37 °C. The bacterial colonies on LB-agar plate were eventually counted. Additionally, 50 μl of 10^8^ CFU ml^−1^ bacterial suspension was added on the blank plate followed by the similar method, which serves as the control group. The antibacterial rate could be calculated from Eq. 4:

$$\text{Antibacterial rate}\;\left(\%\right)=\frac{CFU_{c}-CFU_{s}}{CFU_{c}}\times 100\%$$ (4)

where *CFU*_*c*_ represents the number of bacterial colony of the control group and *CFU*_*s*_ represents the number of bacterial colony of the sample group.

The experiment of dead/live staining was conducted to directly observe the living (green fluorescence) or dead (red fluorescence) state of bacteria using SYTO 9-PI Live and Dead Bacteria Stain Kit. In short, 1 ml of bacteria suspension cocultured with sample was collected and stained for 15 min at 37 °C in the dark by 3 μl of SYTO 9-PI dyes in equal amounts. After that, the bacteria were observed with a fluorescence microscope (IX73, Olympus, Japan). The live/dead staining of bacteria for the control group was observed by the similar method.

### Bacteria-antifouling property

The protein-antifouling property can help to resist the approach of the surface protein of bacteria, which is beneficial to the bacteria-antifouling property. The protein-antifouling properties of samples (DXP@AgNPs, PSAP, and PSAP/DXP@AgNPs) against the model protein of BSA were based on Dong et al.’s [40] protocol. The sample at swelling equilibrium state was immersed in 1 ml of 1 mg ml^−1^ BSA protein solution (in PBS) and incubated for 2 h at 37 °C. Then, the sample hydrogel was washed by PBS, removing free BSA, and soaked in 1 ml of 1 wt% sodium dodecyl sulfate solution for 1 h at ambient temperature to strip the proteins adsorbed on the surface of the hydrogels for detection. The BCA method is utilized to determine the amount of the BSA by detecting the absorbance at 562 nm using a microplate reader. The BSA adsorption capacity is calculated based on the Lambert–Beer law by building the standard curve between absorbance and concentration (Fig. S5). To further evaluate the bacteria-antifouling property of PSAP/DXP@AgNPs against *E. coli* and *S. aureus*, 10 μl of 10^8^ CFU ml^−1^ bacterial suspension was added on surfaces of the PSAP and DXP@AgNPs layers of PSAP/DXP@AgNPs and cocultured for 24 h at 37 °C. In addition, 10 μl of 10^8^ CFU ml^−1^ bacterial suspension was added on the blank plate, serving as the control group. Later, the surfaces of the PSAP and DXP@AgNPs layers of PSAP/DXP@AgNPs were washed by PBS and stained by the mixed SYTO 9-PI dyes for 15 min at 37 °C in the dark. The bacteria in the blank plate were stained by the same way. Finally, the state of bacteria was observed with a fluorescence microscope.

### Biocompatibility

The cytocompatibility was evaluated by employing a leaching pattern test of L929 cells (Zhong Qiao Xin Zhou Biotechnology Co. Ltd., China) for different materials (PSAP, PSAP/DXP, and PSAP/DXP@AgNPs). The L929 cells were cultured to the third generation in complete growth medium containing 81% α-MEM, 10% FBS, and 1% penicillin–streptomycin solution. Then, the cells were seeded into a 96-well plate with the density of 5 × 10^3^ cells per well and incubated in an incubator containing 5% CO_2_ at 37 °C for 24 h. The sterilized leaching solutions of PSAP, PSAP/DXP, and PSAP/DXP@AgNPs were obtained by extraction with PBS for 24 h and then diluted with complete α-MEM at a concentration of 5 mg ml^−1^ as different samples to use. One hundred microliters of 5 mg ml^−1^ sample (with 10% FBS) was cocultured with the L929 cells for 1 day in the incubator. Then, the sample was continued to be cocultured with L929 cells for 3 days. The CCK-8 assay kit was used to measure the cytotoxicity for sample. Briefly, 100 μl of 10 vol% CCK-8 solution relative to the complete α-MEM was directly added to each well and then incubated at 37 °C for 3 h in the dark. The optical density (OD) value was measured at 450 nm by a microplate reader. The cell viability (CV) was calculated according to Eq. 5:

$$\mathrm{CV}\left(\%\right)=\frac{OD_{\text{sample}450}-OD_{\text{blank}450}}{OD_{\text{control}450}-OD_{\text{blank}450}}\times 100\%$$ (5)

where *OD*_*450*_ represents the OD value of the experimental group (cells cocultured with sample) and *OD*_*control*_ and *OD*_*blank*_ represent the OD values of the control group (cells cocultured with complete growth medium) and the blank group (no cells), respectively. The proliferation ratio was calculated according to Eq. 6:

$$P\text{roliferation ratio}\left(\%\right)=\frac{CV_{3d}\left(\%\right)}{CV_{1d}\left(\%\right)}\times 100\%$$ (6)

where *CV*_*3d*_ represents the CV of L929 cells cocultured for 3 days and *CV*_*1d*_ represents the CV of L929 cells cocultured for 1 day.

For the observation of live/dead staining, the L929 cells were cocultured with the complete growth medium for 24 h in a 24-well plate with a density of 2.5 × 10^4^ cells per well. Then, the L929 cells were cocultured with the sample (5 mg ml^−1^) for 1 and 3 days. After that, the mixture of FDA (live cell, green) and PI (dead cell, red) in PBS was utilized to stain L929 cells for 15 min in the dark. The cells were washed with PBS 5 times followed by an observation with a fluorescence microscope (IX73, Olympus, Japan). For the observation of morphological staining, the L929 cells were cocultured with the same procedures as described in live/dead staining. Subsequently, the attached cells were fixed with 4% paraformaldehyde for 15 min, followed by permeabilization with 0.1% Triton X-100 at ambient temperature for 5 min. Then, the cells were stained for 30 min using rhodamine-labeled phalloidin (cytoskeleton, red) in the dark. Afterward, the washed cells were stained for 10 min using DAPI (nucleus, blue) in the dark. Finally, the cells were washed thoroughly by PBS 5 times and observed with a fluorescence microscope.

### Statistical analysis

All data were represented as mean ± standard deviation (SD) calculated from 3 independent experiments. Statistical analysis was conducted by Student’s *t* test or one-way analysis of variance (ANOVA) with Tukey’s multiple comparison test using GraphPad Prism v.8.0 (GraphPad Software).

## Data Availability

Data will be made available on request.

## Supplementary Materials

Supplementary 1 Figs. S1 to S5 Reference
